# 

**Published:** 1891-06

**Authors:** 


### Bristol Medico-Chirurgical Journal.

JUNE, 1891.

### CONTENTS.

ORIGINAL PAPERS: Page

On Modern Methods of Diagnosis in Gastric Affections. By J. MICHELL CLARKE, M.A., M.B., M.R.C.P. ... ... ... ... ... ... ... ... 73

Case of Septic Endocarditis with Cerebral Embolism. By F. H. EDGEWORTH, M.B., B.C., B.A. Cantab., B.Sc. Lond. ... ... ... 89

PROGRESS OF THE MEDICAL SCIENCES ... ... ... ... ... ... ... ... ... 94

REVIEWS OF BOOKS ... ... ... ... ... ... ... ... ... ... ... ... ... ... 119

NOTES ON PREPARATIONS FOR THE SICK ... ... ... ... ... ... ... ... ... 137

MEETINGS ... ... ... ... ... ... ... ... ... ... ... ... ... ... ... 140

VIRCHOW TESTIMONIAL FUND ... ... ... ... ... ... ... ... ... ... 142

SCRAPS ... ... ... ... ... ... ... ... ... ... ... ... ... ... ... 143

